# N-acetylgalatosamine-Mediated Regulation of the *aga* Operon by AgaR in *Streptococcus pneumoniae*

**DOI:** 10.3389/fcimb.2016.00101

**Published:** 2016-09-12

**Authors:** Muhammad Afzal, Sulman Shafeeq, Hifza Ahmed, Oscar P. Kuipers

**Affiliations:** ^1^Department of Molecular Genetics, Groningen Biomolecular Sciences and Biotechnology Institute, University of GroningenGroningen, Netherlands; ^2^Department of Bioinformatics and Biotechnology, Government College University FaisalabadFaisalabad, Pakistan; ^3^Department of Microbiology, Tumor and Cell Biology, Karolinska InstitutetStockholm, Sweden

**Keywords:** N-acetylgalactosamine, AgaR, BgaC, *aga*, CcpA, pneumococcus

## Abstract

Here, we analyze the transcriptomic response of *Streptococcus pneumoniae* D39 to N-acetylgalactosamine (NAGa). Transcriptome comparison of *S. pneumoniae* D39 grown in NAGaM17 (0.5% NAGa + M17) to that grown in GM17 (0.5% Glucose + M17) revealed the elevated expression of various carbon metabolic genes/operons, including a PTS operon (denoted here as the *aga* operon), which is putatively involved in NAGa transport and utilization, in the presence of NAGa. We further studied the role of a GntR-family transcriptional regulator (denoted here as AgaR) in the regulation of *aga* operon. Our transcriptome and RT-PCR data suggest the role of AgaR as a transcriptional repressor of the *aga* operon. We predicted a 20-bp operator site of AagR (5′-ATAATTAATATAACAACAAA-3′) in the promoter region of the *aga* operon (P*bgaC*), which was further verified by mutating the AgaR operator site in the respective promoter. The role of CcpA in the additional regulation of the *aga* operon was elucidated by further transcriptome analyses and confirmed by quantitative RT-PCR.

## Introduction

The human pathogen *Streptococcus pneumoniae* causes a number of infections like pneumonia, sepsis, meningitis, otitis media, and conjunctivitis, and results in over a million of deaths each year worldwide (Ispahani et al., 2004; O'Brien et al., 2009). The genomic abundance of sugar transport genes suggests the importance of carbohydrates in the lifestyle of *S. pneumoniae*. The ability to utilize different nutrient sources plays a vital role in the life-style of the pneumococcus in addition to the virulence factors it possesses (Phillips et al., 1990; Titgemeyer and Hillen, 2002; Carvalho et al., 2011). The prediction of the involvement of over 30% of the transporters in the *S. pneumoniae* genome in the transport of carbohydrates has been authenticated by a recent functional genomic approach targeting carbohydrate transport (Tettelin et al., 2001; Bidossi et al., 2012).

Free carbohydrates are scarce in the human airway, which makes modification and import of complex glycans much more critical for pneumococci (Buckwalter and King, 2012). The human pathogen *S. pneumoniae* encounters glycoconjugates in the nasopharynx, which are composed of a number of monosaccharides and can be used as nutrients after having been depolymerized by glycosidases. The presence of at least nine surface-associated glycosidases to modify host glycans in pneumococcus enhances the chances of its survival in hosts (King et al., 2006; Burnaugh et al., 2008; Dalia et al., 2010). Amino sugars including NAGa (N-acetylgalactosamine) are part of various cell structures in many biological environments. NAGa is a prominent component of the cell wall in bacteria (Freymond et al., 2006), being present in lipopolysaccharides (Bernatchez et al., 2005). Furthermore, NAGa links carbohydrate chains in mucins in humans (Carraway and Hull, 1991). These amino sugars are also generally present in the carbohydrate chains of glycosylated proteins, both in prokaryotes and eukaryotes (Barr and Nordin, 1980; Abu-Qarn et al., 2008). Regulatory mechanisms of a number of carbohydrate and amino acid systems that are vital for the life-style and virulence of *S. pneumoniae*, have been studied (Kloosterman et al., 2006a; Carvalho et al., 2011; Afzal et al., 2015a,b). Successful survival and virulence of *S. pneumoniae* depend on its ability to utilize complex glycans existing at the site of its colonization (Buckwalter and King, 2012; Linke et al., 2013). Its cell envelope is comprised of several layers of peptidoglycan with bound teichoic acids and lipoteichoic acids (LTAs), which are anchored in the cell membrane (Stool and Field, 1989). Pneumococcal LTAs contain phosphocholine and NAGa (Behr et al., 1992). The presence of NAGa in the cell wall of *S. pneumoniae* may indicate its importance as a carbon source for the cell as pneumococcus possesses both secreted and surface-associated glycosidases that may modify glycoconjugates present in the host environment.

The current study demonstrates the impact of NAGa on the transcriptome of *S. pneumoniae* D39 and points to the regulatory mechanism of the *aga* operon. Our transcriptome analysis with D39 Δ*agaR* suggests the role of AgaR as a transcriptional repressor of the *aga* operon. The transcriptome date was further confirmed by RT-PCR analysis. A putative operator site of AgaR in the promoter region of the *aga* operon (P*bgaC*) is predicted and further verified by promoter-mutation studies. Moreover, we demonstrate by transcriptome analysis that the regulation of the *aga* operon is also CcpA-dependent in *S. pneumoniae* D39.

## Materials and methods

### Bacterial strains, growth conditions, and DNA isolation and manipulation

Bacterial strains and plasmids used in this study are listed in Table 1. *S. pneumonia*e D39 was grown as described previously (Kloosterman et al., 2006b; Afzal et al., 2014). For selection on antibiotics, the medium was supplemented with the following concentrations of antibiotics: 2.5 μg/ml tetracycline for *S. pneumoniae*; and 100 μg/ml ampicillin for *Escherichia coli*. All bacterial strains used in this study were stored in 10% (v/v) glycerol at −80°C. All DNA manipulations in this study were done as described before (Shafeeq et al., 2013). For PCR amplification, chromosomal DNA of *S. pneumoniae* D39 (Lanie et al., 2007) was used. Primers used in this study are based on the sequence of the *S. pneumoniae* D39 genome and listed in Table 2.

**Table 1:** **List of strains and plasmids used in this study**.

**Strain/Plasmid**	**Description**	**Source**
***S. PNEUMONIAE***		
D39	Serotype 2 strain. *cps 2*	Laboratory of *P. hermans*
MA900	D39 Δ*agaR*, containing unmarked chromosomal deletion of *agaR*	This study
Δ*ccpA*	D39 Δ*ccpA*; Spec^R^	Carvalho et al., 2011
MA901	D39 Δ*bgaA*::P*bgaC*-*lacZ*; Tet^R^	This study
MA902	D39 Δ*bgaA*::P*bgaC*-*M*-*lacZ*; Tet^R^	This study
MA903	D39 Δ*bgaA*::P*bgaC*-*cre*-*M*-*lacZ*; Tet^R^	This study
MA904	MA900 Δ*bgaA*::P*bgaC*-*cre*-*M*-*lacZ*; Tet^R^	This study
***E. COLI***		
EC1000		Laboratory collection
**PLASMIDS**		
pPP2	Amp^R^ Tet^R^; promoter-less *lacZ*. For replacement of *bgaA* with promoter *lacZ* fusion. Derivative of pPP1	Halfmann et al., 2007
pORI280	ErmR; *ori*^+^ *repA*^−^*;* deletion derivative of pWV01; constitutive *lacZ* expression from P32 promoter	Leenhouts et al., 1998
pMA901	pPP2 P*bgaC-lacZ*	This study
pMA902	pPP2 P*bgaC-M-lacZ*	This study
pMA903	pPP2 P*bgaC-cre*-*M-lacZ*	This study
pMA904	pORI280 carrying *agaR* deletion	This study

**Table 2:** **List of primers used in this study**.

**Name**	**Nucleotide sequence (5′3′)^a^**	**Restriction site**
bgaC-R	CATGGGATCCCTGTGATAGAGCTGACATCG	BamHI
bgaC-F	CATGGAATTCGATACCAATCCTCTGGAGG	EcoRI
bgaC-M-F	CATGGAATTCTTCTATTGACAATTCAAACAGATTGGTTTATAATTAAGCGCACAACAAATG	EcoRI
bgaC-M-ccpA-F	CATGGAATTCCAGATTGGTTTATAATTAATATAACAACAAATGACCGCGCAAACTTTCG	EcoRI
agaR_KO_1	CATGGGATCCGATACCAATCCTCTGGAGG	BamHI
agaR_KO_2	CTGTGATAGAGCTGACATCG	–
agaR_KO_3	GTCAGCTCTATCACAGCATCTCATGTGACCGTGATC	–
agaR_KO_4	CATGGAATTCGTAGGAGTAACTCCATCGG	EcoRI
**QUANTITATIVE RT-PCR PRIMERS**		
63-q1	GCCTTCCAAGCACAGAC	–
63-q2	GGTGTTTCCTCAGTCCG	–
65-q1	CGAGCCTCGTGAAGGTGAG	–
65-q2	GCTGGGTCGGATGAGCG	–
66-q1	GGATGCCGTATTGATGGAC	–
66-q2	GGTGGTGTCGCAAGTTTC	–
67-q1	GGGAGATGTGACTACTGG	–
67-q2	GCTGTCGCAAGAACCGCACC	–
68-q1	GGTTGGAACTACGAACG	–
68-q2	CCAGCGATAATGGTATGG	–
69-q1	CACAGTAGCTCTTCTTCC	–
69-q2	CCATTGGAAGATTCATCCC	–
71-q1	GGCGAGGATGTCTTGGC	–
71-q2	CCTACACTTGCTCCATGC	–
**RT PCR PRIMERS**		
IR-I-1	GGAAGGTGCCAACCGTATC	–
IR-I-2	CGTCGTCTACAACCATAATGC	–
IR-II-1	CGTTCTATCAACGTAGTAG	–
IR-II-2	CCTGCAGATGAAACGATCG	–
IR-III-1	GCTGTAGCAGCACCTTCTAC	–
IR-III-2	CCAGAAGCTTGCATACGTTCG	–
IR-IV-1	GCTATCGGTATTATTATCG	–
IR-IV-2	CAGCTTCAAATTTAGCAG	–
IR-V-1	GCGGGCTTCGATGATGACG	–
IR-V-2	GCACCCAATTCGAGCAAGTC	–
IR-V-1	CCGTGTAGTACAAGGTGTC	–
IR-V-2	GCCAAGACATCCTCGCCCTC	–

a *Restriction enzyme sites are underlined*.

### Construction of the *agaR* mutant

A marker-less *agaR* mutant (Δ*agaR*: MA900) was constructed in *S. pneumoniae* D39 using the pORI280 plasmid, as described before (Kloosterman et al., 2006b). The primer pairs, agaR-1/agaR-2 and agaR-3/agaR-4, were used to generate PCR fragments of the left and right flanking regions of *agaR* (each of nearly 600 bp), respectively. The left and right flanking regions contain BamHI and EcoRI restriction sites as have the pORI280. These PCR fragments were inserted into pORI280 using these restriction sites, and the construct (pMA904) was used to transform *S. pneumoniae* D39 with selection for erythromycin resistance. The transformation leads to single cross-over integration of the construct into the chromosome, as pORI280 depends on RepA for replication. Several erythromycin-resistant, LacZ-positive integrants, as separate cultures for 30–50 generations (culturing two to four times until stationary phase) without antibiotic selection were plated on X-Gal medium. Due to this, we could screen for clones that had lost the integration due to a second recombination event. 0.5% of the colonies were both white and erythromycin sensitive, signifying excision of the plasmid from the chromosome. Eighty percent of these white erythromycin-sensitive colonies had the desired mutation, as verified by PCR and DNA sequencing.

### Construction of promoter *lacZ*-fusions and β-galactosidase assays

The chromosomal transcriptional *lacZ*-fusion to the *bgaC* promoter was constructed in the integration plasmid pPP2 (Halfmann et al., 2007) *via* double crossover in the *bgaA* locus of *S. pneumoniae* D39 with the primer pairs mentioned in Table 2 (bgaC-R and bgaC-F), resulting in pMA901. P*bgaC*-*M*-*lacZ* (mutation in AgaR operator site) was constructed in pPP2 (Halfmann et al., 2007), using the primer pairs mentioned in Table 2 (bgaC-M-F and bgaC-R), resulting in plasmid pMA902. These constructs were further introduced into the *S. pneumoniae* D39 wild-type resulting in strains MA901 and MA902, respectively. Similarly, P*bgaC*-*M*-*ccpA*-*lacZ* (mutation in *cre* site) was constructed in pPP2 (Halfmann et al., 2007), using the primer pairs mentioned in Table 2, resulting in plasmid pMA903. These constructs were further introduced into the *S. pneumoniae* D39 wild-type and D39 Δ*agaR* (MA900) resulting in strains MA903 and MA904, respectively. All plasmid constructs were checked by PCR and DNA sequencing.

β-galactosidase assays were performed as described before (Israelsen et al., 1995; Kloosterman et al., 2006b) using cells that were harvested in the mid-exponential phase of growth, grown in M17 medium with appropriate sugars mentioned in Results Section. M17 medium is composed of pancreatic digest of casein, soy peptone, beef extract, yeast extract, and minerals.

### RNA extraction, reverse transcription (RT)-PCR, and purification for quantitative RT-PCR

Total RNA was isolated from *S. pneumoniae* D39 wild-type and D39 Δ*agaR* (MA900) grown in GM17 (0.5% Glucose + M17) as described (Shafeeq et al., 2011a). Similarly, total RNA was isolated from *S. pneumoniae* D39 wild-type and D39 Δ*ccpA* grown in NAGaM17 (0.5% NAGa + M17) as described (Shafeeq et al., 2011a). The RNA sample was treated with 2U of RNase free Dnase I (Invitrogen, Paisley, UK) to remove any DNA contamination. First, strand cDNA synthesis was performed on RNA (Shafeeq et al., 2011a). cDNA (2 μl) was amplified in a 20 μl reaction volume that contained 3 pmol of each primer and the reactions were performed in triplicate (Shafeeq et al., 2011a). The transcription level of specific genes was normalized to *gyrA* transcription, and amplified in parallel with the gyrA-F and gyrA-R primers. The results were interpreted using the comparative CT method (Schmittgen and Livak, 2008).

To confirm that the *aga* operon is transcribed as a single transcriptional unit, *S. pneumoniae* D39 wild-type was grown in NAGaM17 (0.5% NAGa + M17) and total RNA was isolated as described (Shafeeq et al., 2011a). RT-PCR (reverse transcription PCR) was performed as described before (Afzal et al., 2014) on all possible intergenic regions of the *aga* operon with primer pairs mentioned in Table 2. For a fair comparison of the PCR products, 100 ng of RNA and 20 ng of DNA were used.

### Microarray analysis

For DNA microarray analysis in the presence of NAGa, the transcriptome of *S. pneumoniae* D39 wild-type, grown in biological replicates in GM17 (0.5% Glucose + M17) and NAGa (0.5% NAGa + M17) were compared. For DNA microarray analysis of D39 Δ*agaR* (MA900), the transcriptome of *S. pneumoniae* D39 wild-type and D39 Δ*agaR*, grown in biological replicates in GM17 (0.5% Glucose + M17) was compared. Similarly, for DNA microarray analysis of D39 Δ*ccpA*, the transcriptome of *S. pneumoniae* D39 wild-type and D39 Δ*ccpA*, grown in biological replicates in NAGaM17 (0.5% NAGa + M17) was compared. The cells were harvested at their respective mid-exponential growth phases. All other procedures regarding the DNA microarray experiment and data analysis were performed as previously described (Afzal et al., 2015a; Shafeeq et al., 2015). Briefly, microarray slide images were scanned using GenPix Pro 6.1 (MSD analytical technologies). Processing and normalization (LOWESS spotpin-based) of slides were performed with the in-house developed MicroPrep software. DNA microarray data were obtained from independent biological replicates hybridized to glass slides, of which one was a dye swap. Expression ratios were calculated from the measurements of at least five spots. Differential expression tests were performed on expression ratios with a local copy of the Cyber-T implementation of a variant of the *t*-test. For the identification of differentially expressed genes a Bayesian *p*-value of < 0.001 and a fold change cut-off 2 was applied. Microarray data have been submitted to GEO under accession number GSE86008.

## Results

### NAGa-dependent gene expression in *S. pneumoniae*

To study the response of *S. pneumoniae* D39 to NAGa, we preformed transcriptome comparison of *S. pneumoniae* D39 wild-type grown in NAGaM17 (0.5% NAGa + M17) to that in glucose (0.5% Glucose + M17). Table 3 summarizes the transcriptome changes incurred on *S. pneumoniae* in the presence of NAGa. The presence of NAGa in the medium resulted in the upregulation of a number of carbon metabolic genes/operons after applying the criteria of ≥2-fold difference and a *p*-value of < 0.001.

**Table 3:** **Summary of transcriptome comparison of ***S. pneumoniae*** D39 wild-type grown in NAGaM17 (0.5% NAGa + M17) to that grown in GM17 (0.5% Glucose + M17)**.

**D39 tag^a^**	**Function^b^**	**Ratio^c^**
*spd_0030*	Hypothetical protein	−7.6
*spd_0031*	Hypothetical protein	−4.1
*spd_0032*	Hypothetical protein	−3.1
*spd_0065*	Beta-galactosidase, BgaC	2.0
*spd_0066*	PTS system, IIB component, GadV	3.6
*spd_0067*	PTS system, IIC component, GadW	3.5
*spd_0068*	PTS system, IID component, GadE	3.7
*spd_0069*	PTS system, IIA component, GadF	3.3
*spd_0070*	Sugar isomerase domain protein, AgaS	6.4
*spd_0071*	Aldose 1-epimerase, GalM	2.0
*spd_0088*	ABC transporter, permease protein	5.9
*spd_0089*	ABC transporter, permease protein	6.4
*spd_0090*	ABC transporter, substrate-binding protein	76.8
*spd_0091*	Hypothetical protein	−5.4
*spd_0092*	Hypothetical protein	−6.5
*spd_0447*	Transcriptional regulator, MerR family protein, GlnR	−4.1
*spd_0448*	Glutamine synthetase, type I, GlnA	−2.0
*spd_0490*	Hypothetical protein	−4.3
*spd_0553*	Hypothetical protein	−7.1
*spd_0559*	PTS system IIA component, putative	5.0
*spd_0560*	PTS system, IIB component, putative	5.7
*spd_0561*	PTS system, IIC component, putative	5.4
*spd_0608*	Orotidine 5'-phosphate decarboxylase, PyrF	−48.0
*spd_0609*	Orotate phosphoribosyltransferase, PyrE	−39.5
*spd_0675*	Hypothetical protein	−5.5
*spd_0731*	DNA topology modulation protein FlaR, putative	−31.3
*spd_0850*	Lactoylglutathione lyase	−19.0
*spd_0851*	Dihydroorotate dehydrogenase electron transfer subunit, PyrK	−66.2
*spd_0852*	Dihydroorotate dehydrogenase, catalytic subunit, PyrDb	−32.5
*spd_1050*	Tagatose 1,6-diphosphate aldolase, LacD	29.6
*spd_1051*	Tagatose-6-phosphate kinase, LacC	15.3
*spd_1052*	Galactose-6-phosphate isomerase, LacB subunit	20.2
*spd_1053*	Galactose-6-phosphate isomerase, LacA subunit	19.4
*spd_1133*	Aspartate carbamoyltransferase, PyrB	−18.2
*spd_1134*	Pyrimidine operon regulatory protein/uracil phosphoribosyltransferase, PyrR	−26.0
*spd_1427*	PhnA protein	−11.4
*spd_1489*	N-acetylneuraminate lyase, NanA2	3.7
*spd_1490*	Hypothetical protein	3.5
*spd_1491*	Hypothetical protein	7.4
*spd_1492*	Hypothetical protein	3.9
*spd_1493*	Sugar ABC transporter, permease protein, NanW	4.8
*spd_1494*	Sugar ABC transporter, permease protein, NanV	2.5
*spd_1495*	Sugar ABC transporter, sugar-binding protein, NanU	6.4
*spd_1496*	PTS system, IIBC components, NanP	3.2
*spd_1497*	N-acetylmannosamine-6-phosphate 2-epimerase 2, NanE	3.1
*spd_1504*	Sialidase A, NanA	16.6
*spd_1633*	Galactose-1-phosphate uridylyltransferase, GalT2	21.6
*spd_1634*	Galactokinase, GalK	27.6
*spd_1635*	Galactose operon repressor, GalR	5.5
*spd_1726*	Pneumolysin, Ply	−4.0
*spd_1866*	N-acetylglucosamine-6-phosphate deacetylase, NagA	3.7
*spd_1898*	Hypothetical protein	−3.0
*spd_1899*	Glutamine amidotransferase, class 1, YvdE	−3.4
*spd_1969*	Glycosyl hydrolase-related protein	12.3
*spd_1970*	ROK family protein	31.2
*spd_1971*	Glycosyl hydrolase-related protein	5.1
*spd_1972*	Hypothetical protein	6.8
*spd_1973*	Alpha-1,2-mannosidase, putative	2.7
*spd_1974*	Hypothetical protein	4.0

a *Gene numbers refer to D39 locus tags*.

b *D39 annotation (Lanie et al., 2007)*.

c *Ratio represents the fold increase/decrease in the expression of genes in NAGa compared to glucose. All p-values were < 0.001*.

A system for sialic acid transport and utilization is upregulated in the presence of NAGa. This system consists of a neuraminidase (*nanA*) and *nan* operon-I (Afzal et al., 2015c). *nanA* has been demonstrated to be involved in virulence of *S. pneumoniae* (Dalia et al., 2010). Another important gene coding for an N-acetylglucosamine-6-phosphate deacetylase (NagA) is also upregulated in the presence of NAGa. N-acetylglucosamine (NAG) is phosphorylated by the PTS and this phosphorylated N-acetylglucosamine is deacetylated to glucosamine-6-P by NagA (Kanehisa et al., 2014). Another putative operon *spd_1969-72* is highly upregulated in our microarray analysis in the presence of NAGa. This putative operon is supposedly involved in the utilization of amino sugars and in the conversion of chitobiose into NAG (Kanehisa et al., 2014). Further experiments are needed to explore the role of this operon in the utilization of amino sugars.

An ABC transporter (encoded by *spd-0088-90*) putatively involved in galactose transport (Bidossi et al., 2012) is among the ones upregulated in our NAGa transcriptome analysis. Furthermore, Leloir pathway genes (*galKT*) (Afzal et al., 2015d) are highly upregulated in the presence of NAGa. Galactose can also be utilized through the Tagatose pathway and we could observe significant upregulation of the Tagatose pathway genes (*lacABCD*) in the presence of NAGa. The Tagatose pathway genes are present in an operon (*lac* operon-I) and are involved in the utilization of lactose and galactose (Afzal et al., 2014). The expression of a number of other genes putatively involved in the transport and utilization of carbohydrates are also altered in the presence of NAGa. The altered expression of these genes might be due to the absence of CCR (carbon catabolite repression) in the presence of NAGa. We have analyzed the promoter regions of these genes and we could find putative *cre* boxes in the promoter regions of these genes.

*glnAR* genes that are part of the glutamine regulon were downregulated in the presence of NAGa. This regulon consists of genes involved in glutamine synthesis and uptake (*glnA* and *glnPQ*), glutamate synthesis (*gdhA*), and the gene coding for the pentose phosphate pathway enzyme Zwf, which forms an operon with *glnPQ* (Kloosterman et al., 2006a). The glutamine regulon is shown to be repressed in the presence of a nitrogen source (Kloosterman et al., 2006a). The down-regulation of the glutamine regulon might be due to the presence of nitrogen in NAGa.

The expression of genes that are putatively involved in NAGa transport and utilization (the putative *aga* gene cluster) was also altered in our transcriptome analysis. The upregulation of the *aga* gene cluster might indicate that the system for putative transport and utilization of NAGa is functional and responds to NAGa in *S. pneumoniae*. Therefore, we decided to further characterize the *aga* gene cluster in *S. pneumoniae* D39.

### Organization of the *aga* gene cluster in *S. pneumoniae*

**Figure 1:**
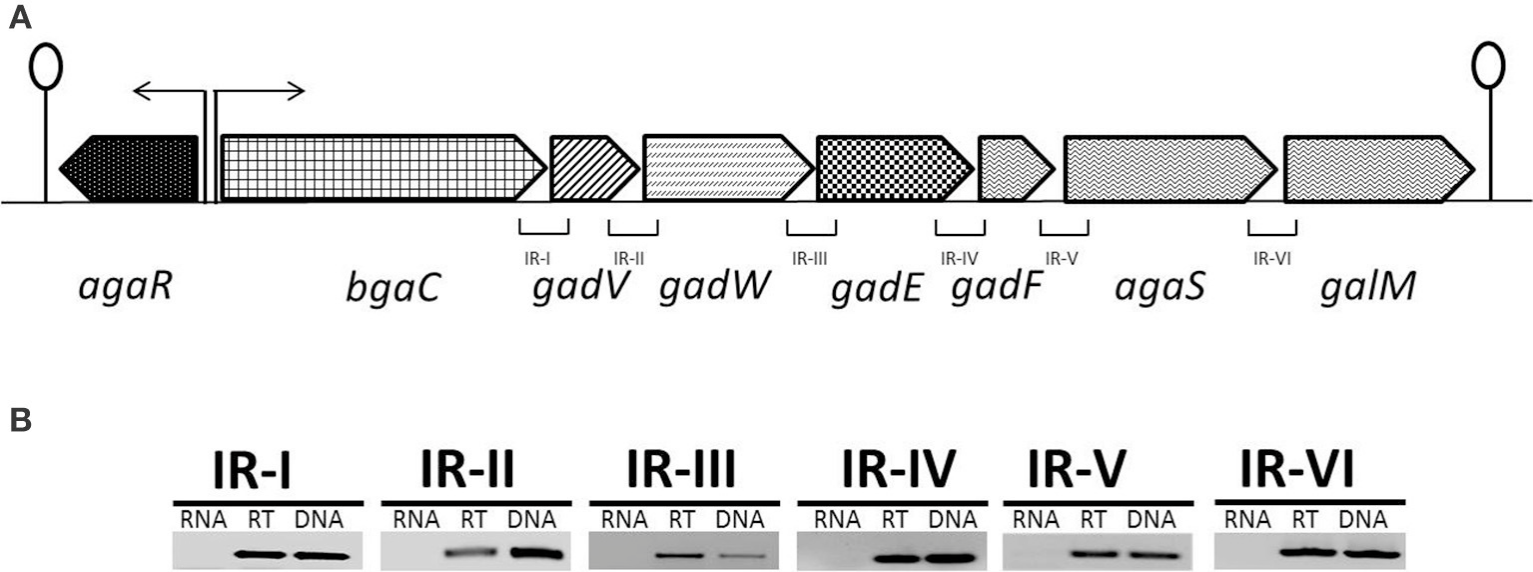
**(A)** Organization of the *aga* operon in *S. pneumoniae* D39. A lollipop structure represents a putative transcriptional terminator, while black arrows indicate the promoter regions. See text for further details. **(B)** Reverse transcriptase (RT)-PCR analysis to confirm the polycistronic nature of the *aga* operon in *S. pneumoniae* D39. RT-PCR was performed on total RNA isolated from *S. pneumoniae* D39 wild-type grown in NAGaM17 (0.5% NAGa + M17) medium with (RT) and without (RNA) reverse transcriptase treatment using the intergenic region primer pairs. DNA was used as a positive control. The size of the RT-PCR products ranges from 100 to 300 bp.

The putative *aga* gene cluster consists of seven genes encoding a beta-galactosidase (*bgaC*), a predicted galactosamine disaccharide-specific PTS system (IIBCDA components: *gadVWEF*), a gene coding for a sugar isomerase (*agaS*) and a gene coding for an aldose 1-epimerase (*galM*) (Figure 1A). To confirm whether the putative *aga* gene cluster transcribes as a single transcriptional unit, we performed RT-PCR on all possible intergenic regions present in the *aga* gene cluster with the primer pairs mentioned in Table 2. RT-PCR data revealed that the putative *aga* gene cluster transcribes as a single transcriptional unit (Figure 1B) and from here on, we call it the *aga* operon.

### NAGa induces the expression of the *aga* operon

**Figure 2:**
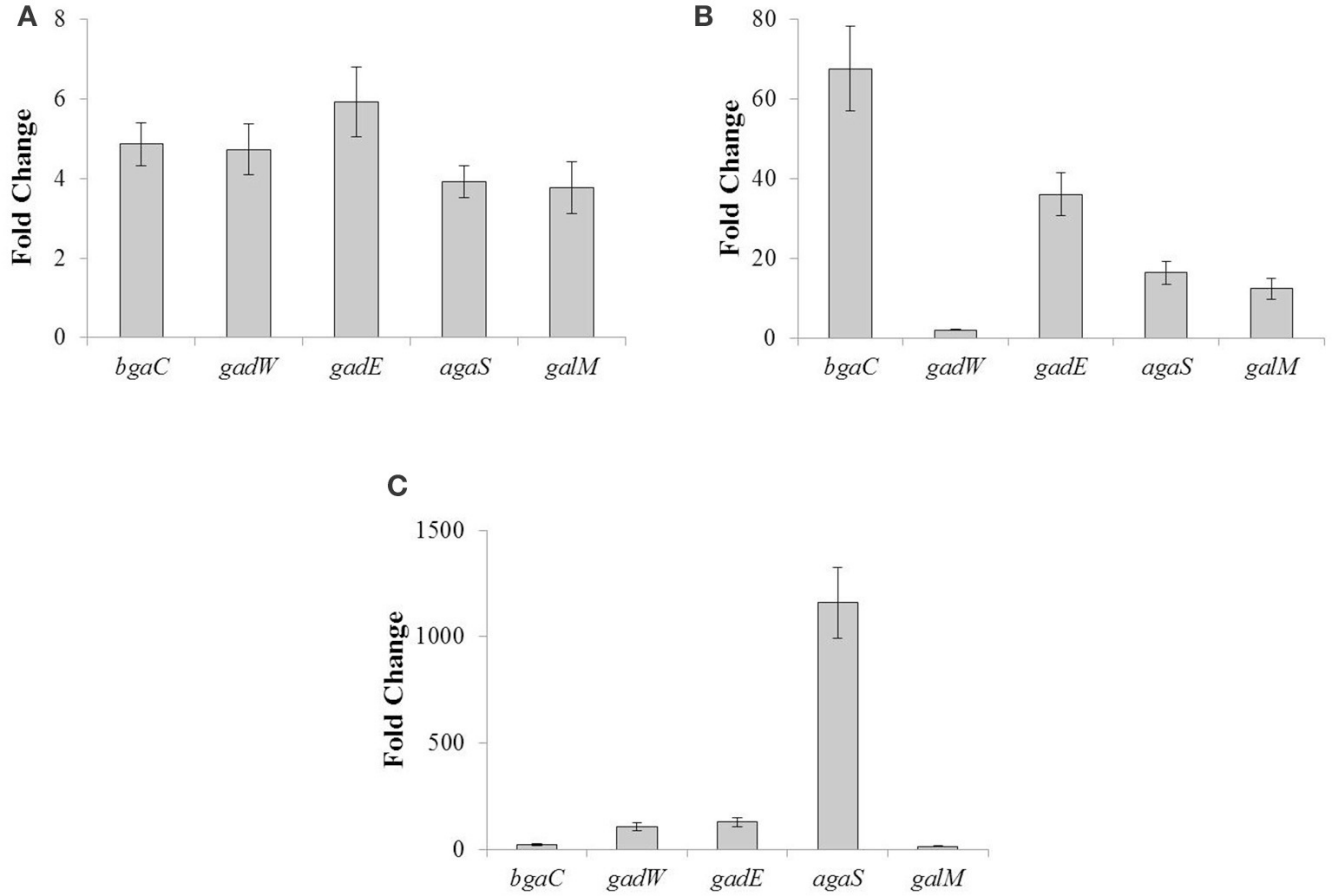
**The relative increase in the expression of the genes belonging to the ***aga*** operon**. **(A)** *S. pneumoniae* D39 wild-type was grown in GM17 (0.5% Glucose + M17) and NAGaM17 (0.5% NAGa + M17). Ratio represents an increase in the expression of the *aga* operon genes in *S. pneumoniae* D39 wild-type grown in NAGaM17 to the one grown in GM17. **(B)** *S. pneumoniae* D39 wild-type and D39 Δ*agaR* both grown in GM17 (0.5% Glucose + M17). Ratio represents an increase in the expression of the *aga* operon genes in *S. pneumoniae* D39 Δ*agaR* compared to D39 wild-type. **(C)** *S. pneumoniae* D39 wild-type and D39 Δ*ccpA* both grown in NAGaM17 (0.5% NAGa + M17). Ratio represents an increase in the expression of the *aga* operon genes in *S. pneumoniae* D39 Δ*ccpA* compared to D39 wild-type. The expression of the *aga* genes was normalized with housekeeping gene *gyrA*. Results represent the mean and standard deviation of three independent replications.

To further confirm our microarray results and study the role of NAGa in the regulation of the *aga* operon, we grew the *S. pneumoniae* D39 wild-type in GM17 (0.5% Glucose + M17) and NAGaM17 (0.5% NAGa + M17) and performed quantitative RT-PCR on the genes of the *aga* operon. The results of quantitative RT-PCR showed that the expression of the *aga* operon increased significantly when grown in the presence of NAGa (Figure 2A). These results further confirm our microarray data mentioned above and demonstrate that the *aga* operon is functional and responds to NAGa in *S. pneumoniae* D39.

### Microarray analysis of D39 *ΔagaR*

AgaR, a GntR-family transcriptional regulator, is encoded by the gene present upstream of the *aga* operon (Figure 1). The presence of the *agaR* gene next to the *aga* operon might indicate its role in the regulation of the *aga* operon. To study whether AgaR is involved in the regulation of the *aga* operon, we constructed a marker-less mutant of *agaR* and performed microarray analysis of D39 Δ*agaR* against D39 wild-type grown in GM17 (0.5% Glucose + M17). GM17 was used as a growth medium as we got repression of the *aga* operon in the presence of glucose. Table 4 summarizes the results of the transcriptomic changes induced in *S. pneumoniae* due to the deletion of *agaR. agaR* was downregulated 26-fold in our transcriptome analysis, confirming *agaR* deletion in Δ*agaR*. After choosing the criterion of ≥2-fold difference as the threshold change and a *p*-value < 0.001, the *aga* operon was upregulated significantly in Δ*agaR* and no other larger responses were observed in the transcriptome. This data further suggests that AgaR is a negative transcriptional regulator of the *aga* operon in the absence of NAGa.

**Table 4:** **Summary of transcriptome comparison of ***S. pneumoniae*** D39 wild-type with D39 Δ***agaR*** grown in GM17 (0.5% Glucose + M17) and ***S. pneumoniae*** strain D39 wild-type with Δ***ccpA*** grown in NAGaM17 (0.5% NAGa + M17)**.

**D39 tag^a^**	**Function^b^**	**Ratio^c^**	**Ratio^d^**
*spd_0064*	GntR-family transcriptional regulator, AgaR	−26.0	−
*spd_0065*	Beta-galactosidase, BgaC	10.9	20.5
*spd_0066*	PTS system, IIB component, GadV	10.8	13.9
*spd_0067*	PTS system, IIC component, GadW	5.9	−
*spd_0068*	PTS system, IID component, GadE	8.0	25.2
*spd_0069*	PTS system, IIA component, GadF	8.5	13.5
*spd_0070*	Sugar isomerase domain protein, AgaS	20.5	5.6
*spd_0071*	Aldose 1-epimerase, GalM	3.4	21.8

a *Gene numbers refer to D39 locus tags*.

b *D39 annotation (Lanie et al., 2007)*.

c *Ratio represents the fold increase/decrease in the expression of genes in D39 ΔagaR compared to the D39 wild-type*.

d *Ratio represents the fold increase/decrease in the expression of genes in D39 ΔccpA compared to the D39 wild-type. The ratios are obtained through microarrays analysis*.

### AgaR acts as a transcriptional repressor of the *aga* operon

To confirm our microarray results of D39 Δ*agaR*, we grew *S. pneumoniae* D39 wild-type and D39 Δ*agaR* in GM17 (0.5% Glucose + M17) and performed quantitative RT-PCR on the genes belonging to the *aga* operon. The results of quantitative RT-PCR showed that the expression of the *aga* operon increased significantly in D39 Δ*agaR* even in the presence of glucose (Figure 2B). These quantitative RT-PCR results further confirm that AgaR represses the expression of the *aga* operon and that this repression is relieved in the absence of *agaR*.

### Prediction and confirmation of the agaR operator site in the promoter region of the *ag*a operon (*bgaC*)

**Figure 3:**
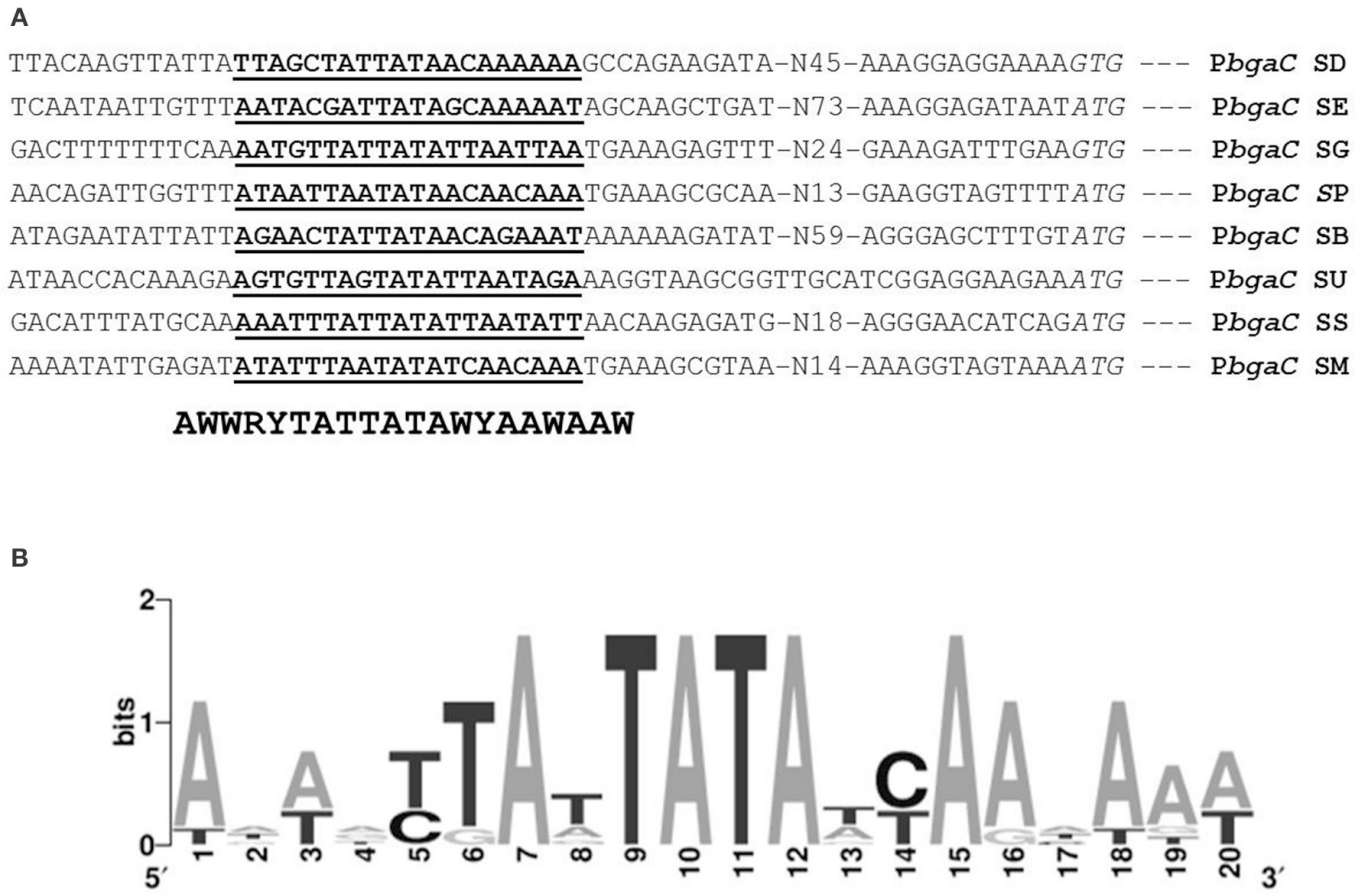
**Identification of the AgaR operator site in P***bgaC***. (A)** Position of the AgaR operator site in P*bgaC* of different streptococci. Translational start sites are italicized and putative AgaR operator sites are bold-underlined. **(B)** Weight matrix of the identified AgaR operator site in the P*bgaC* of different streptococci. SD, *S. dysgalactiae*; SE, *S. equi*; SG, *S. gordonii*; SP, *S. pneumonia*; SB, *S. uberis*; SU, *S. suis*; SS, *S. sanguis;* and SM, *S. mitis*.

AgaR, a putative GntR-family transcriptional regulator, is present next to the *aga* operon in *S. pneumoniae* D39. Using Genome2D tool (Baerends et al., 2004) and a MEME motif sampler search (Bailey and Elkan, 1994), a 20-bp palindromic sequence was found upstream of *bgaC* (5′-ATAATTAATATAACAACAAA-3′) in *S. pneumoniae* D39 wild-type (SP) (Figure 3A). This DNA stretch may serve as an AgaR operator site in *S. pneumoniae*. NAGa genes promoters of other streptococcal species were studied to check if the AgaR operator site is also conserved in those streptococci. The outcome of this analysis was the finding that the AgaR operator sequence is highly conserved in these streptococci as well (Figure 3B). A genome-wide search with the pneumococcal AgaR operator site was conducted to locate more putative AgaR targets in the *S. pneumoniae* D39 genome. We could not find any other DNA stretch similar to the AgaR operator site, which suggests that the *aga* operon is the only target of AgaR.

**Figure 4:**
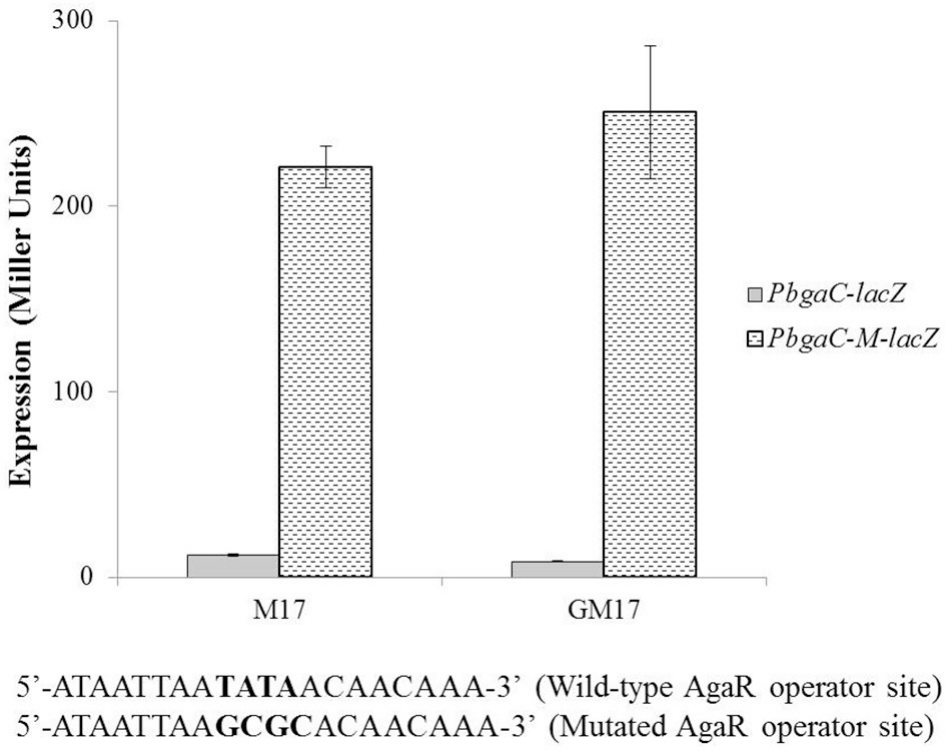
**Expression levels (in Miller units) of P***bgaC-lacZ*** and P***bgaC-M-lacZ*** in D39 wild-type grown in M17 (with no added sugar) and GM17 (0.5% Glucose + M17)**. Standard deviations of three independent experiments are indicated in bars. Wild-type and mutated AgaR operator sites in P*bgaC* are given below.

To determine whether the located stretch of DNA mediates the AgaR-dependent transcriptional control of the *aga* operon, we made a *lacZ*-fusion, where conserved bases in the putative AgaR site were mutated in P*bgaC* (5′-ATAATTAA**TATA** ACAACAAA-3′ to 5′-ATAATTAA**GCGC**AC AACAAA-3′) and performed β-galactosidase assays (Figure 4). The expression of the promoter increased significantly when we mutated a few of the conserved bases of the putative AgaR operator site. These data suggest that the putative AgaR operator site is active and performs the role of AgaR operator site in *S. pneumoniae*. This operator site might also be active in other streptococcal species as it is highly conserved in those species as well.

### The role of CcpA in the regulation of the *aga* operon

The *aga* operon was only around 3-fold upregulated in our transcriptome in the presence of NAGa. The possible reason could be the involvement of another transcriptional regulator that is repressing the *aga* operon in the presence of NAGa. The other transcriptional regulator could be CcpA. CcpA (Carbon catabolite protein A) is the principal transcriptional regulator in *S. pneumoniae* that represses the expression of genes involved in the consumption of non-preferred sugars in the presence of a favored one and has a role in virulence as well (Carvalho et al., 2011). CcpA represses the transcription of genes involved in the utilization non-preferred sugars in the presence of a preferred one by binding to specific DNA sequences called *cre* (Catabolite Repression Element) boxes. To study the role of CcpA in the regulation of the *aga* operon, we analyzed the P*bgaC* for the presence of a putative *cre* box. We could find a *cre* box in the promoter region of *bgaC* (5′-ATGAAAGCGCAAACTT-3′), which might suggest a role of CcpA in the regulation of the *aga* operon.

To determine the role of CcpA in the regulation the *aga* operon, we performed microarray analysis of D39 Δ*ccpA* against the D39 wild-type grown in NAGaM17 (0.5% NAGa + M17). A number of genes were significantly affected in D39 Δ*ccpA* compared to the D39 wild-type in the presence of NAGa. We could observe a significant upregulation in the expression of the *aga* operon in our transcriptome analysis (Table 4), which suggests that CcpA represses the expression of the *aga* operon in the presence of NAGa. These results are in accordance with the data presented in a previous study, where they performed microarray analysis of D39 Δ*ccpA* in the presence of glucose and galactose (Carvalho et al., 2011). There were also a number of other genes/operons that were differentially expressed in D39 Δ*ccpA* in the presence of NAGa. These genes have been grouped into COG functional categories according to the putative function of respective proteins (Table 5). Genes belonging to the category G are mostly carbohydrate transport and metabolism genes and the repression on genes caused by CcpA is relieved in the absence of CcpA.

**Table 5:** **Number of genes significantly affected in D39 Δ***ccpA*** compared to the D39 wild-type grown in NAGaM17 (0.5% NAGa + M17)**.

**Functional categories**	**Total**	**Up**	**Down**
C: Energy production and conversion	21	5	16
E: Amino acid transport and metabolism	25	3	22
F: Nucleotide transport and metabolism	13	3	10
G: Carbohydrate transport and metabolism	45	34	11
H: Coenzyme transport and metabolism	6	1	5
I: Lipid transport and metabolism	4	0	4
J: Translation, ribosomal structure and biogenesis	41	3	38
K: Transcription	10	6	4
L: Replication, recombination and repair	7	0	7
M: Cell wall/membrane/envelope biogenesis	12	5	7
O: Posttranslational modification, protein turnover, chaperones	7	2	5
P: Inorganic ion transport and metabolism	9	1	8
Q: Secondary metabolites biosynthesis, transport and catabolism	2	1	1
R: General function prediction only	25	6	19
S: Function unknown	22	14	8
T: Signal transduction mechanisms	12	5	7
U: Intracellular trafficking, secretion, and vesicular transport	2	0	2
V: Defense mechanisms	6	2	4
Others	45	20	25
Total number of genes	314	111	203

*Genes affected more than 2-fold in D39 ΔccpA as compared to the D39 wild-type are shown in COG functional categories*.

To further confirm our microarray results, we performed quantitative RT-PCR on the genes of the *aga* operon in D39 Δ*ccpA* in NAGaM17 (0.5% NAGa + M17). The results of quantitative RT-PCR show that the expression of the *aga* operon increased significantly in D39 Δ*ccpA* when grown in the presence of NAGa (Figure 2C). These results further confirm that the *aga* operon is repressed by CcpA in *S. pneumoniae* D39.

**Figure 5:**
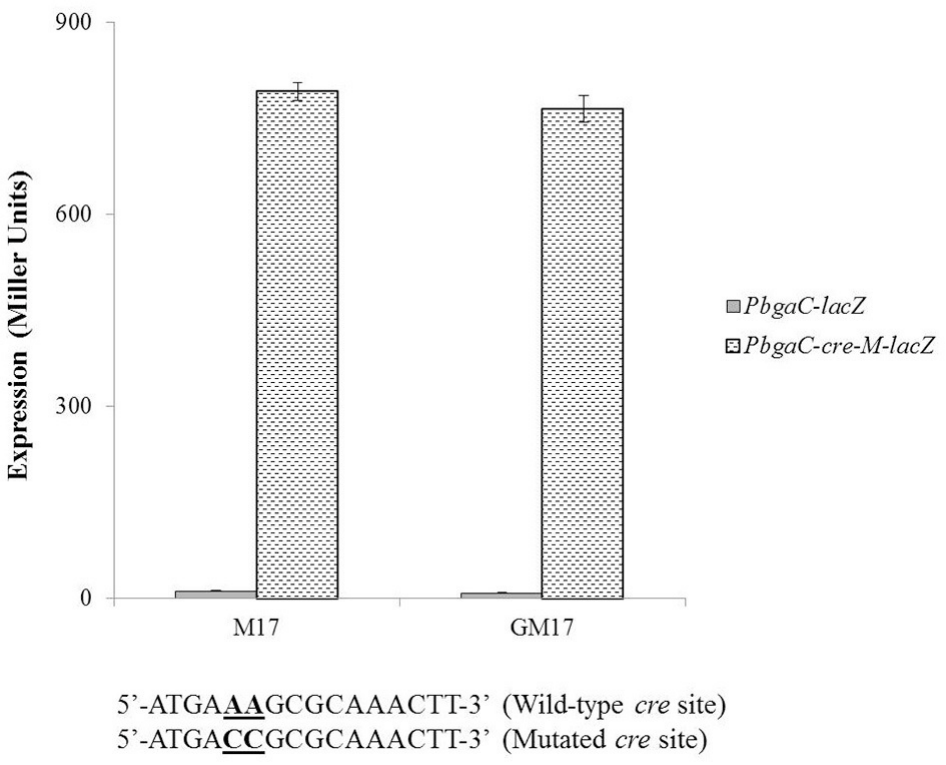
**Expression levels (in Miller units) of P***bgaC-lacZ*** and P***bgaC-cre-M-lacZ*** in D39 wild-type grown in M17 (with no added sugar) and GM17 (0.5% Glucose + M17)**. Standard deviations of three independent experiments are indicated in bars. Wild-type and mutated *cre* sites in P*bgaC* are given below.

To check the functionality of the *cre* site present in promoter region of the *aga* operon, we made a promoter *lacZ*-fusion of *bgaC* (P*bgaC*-*cre*-*M*-*lacZ*) with mutations in the conserved bases of the putative *cre* box (5′-ATGA**AA**GCGCAAACTT-3′ to 5′-ATGA**CC**GCGCAAACTT-3′) and transformed it into D39 wild-type and D39 Δ*agaR*, and performed β-galactosidase assays (Figure 5). The expression of the mutated promoter was significantly higher in the *agaR* mutant, which also confirms the functionality of the predicted *cre* box in the promoter region of *bgaC* and the role of CcpA in regulation of *aga* operon.

## Discussion and conclusions

Extensive studies regarding regulatory mechanisms of different dedicated systems for carbon sources in *S. pneumoniae* emphasize the importance of carbohydrates in the life style of pneumococcus, which confers an extra advantage in its survival in ever changing nutritional environment (Tettelin et al., 2001; Iyer and Camilli, 2007; Afzal et al., 2014, 2015c,d,e). In bacteria, NAGa is a vital constituent of the cell wall as it is present in lipopolysaccharides (Bernatchez et al., 2005; Freymond et al., 2006). NAGa links carbohydrate chains in mucins in humans (Carraway and Hull, 1991). An important aspect of the interaction between the pneumococcus and its human host is the ability of this bacterium to process host glycans. The current study demonstrates the NAGa-dependent gene expression and regulatory mechanism of the *aga* operon in *S. pneumoniae*.

NAGa can support growth of different bacteria, including *E. coli*, acting as a carbon and nitrogen source (Reizer et al., 1996). A NAGa utilization system (AgaBCD and AgaVWEF) was identified in *E. coli* (Reizer et al., 1996). *agaBCD* and *agaVWEF* encode two PTSs that mediate the transport and phosphorylation of galactosamine and NAGa, respectively, in *E. coli* (Brinkkötter et al., 2000). The proposed catabolic pathway of NAGa in *E. coli* involves the transport and subsequent phosphorylation of NAGa, the deacetylation of NAGa-6-P, the deamination/isomerization of GalN-6-P, the phosphorylation of Tag-6-P, and the cleavage of Tag-1,6-PP to produce glyceraldehyde 3-phosphate and glycerone phosphate (Brinkkötter et al., 2000). Moreover, a wide-ranging diversity in galactosamine/NAGa utilization pathways in Proteobacteria such as *Shewanella* has been proposed (Leyn et al., 2012). Particularly, there is a lot of variability among the first steps of the pathway, whereas the concluding three steps are mostly conserved (Leyn et al., 2012). In *E. coli*, AgaR belonging to the DeoR family of transcriptional factors negatively regulates the expression of *agaZ* and *agaS* involved in the galactosamine/NAGa catabolism pathways (Ray and Larson, 2004; Leyn et al., 2012). The genes encoding galactosamine/NAGa pathways were recognized in 16 genomes signifying four families; *Streptococcaceae, Lactobacillaceae, Enterobacteraceae*, and *Carnobacteriaceae*. *Lactobacillus helveticus* and *Streptococcus pyogenes* possess the minimal number of genes in the reconstructed regulons (Zhang et al., 2015). It is very likely that these organisms cannot utilize NAGa as only one or two genes from the NAGa utilization pathway were found in these organisms. *Streptococcus gordonii* and *Streptococcus mitis* have the minimal gene set that allows them to utilize NAGa. These genes include transcriptional regulator (*agaR*), galactosamine-6-phosphate deaminase/isomerase (*agaS*), glycoside hydrolase (*bgaC*) and PTS (*gadVWEF*) (Zhang et al., 2015). The gene for the tagatose-1,6-diphosphate aldolase (*agaY*) is present in some other well-studied *Streptococcal* genomes. On the contrary, all the investigated *Lactobacilli* lack *agaY*, but have the gene for N-acetylgalactosamine-phosphate deacetylase (*agaA*) (Zhang et al., 2015). *agaA* was recognized only in the *Streptococcus suis* genome among *Streptococcaceae*. The gene for tagatose-6-phosphate kinase (*agaZ*) was found only in *Enterococcus faecalis* and *Carnobacterium* (Zhang et al., 2015). The *aga* operon is also annotated as part of the amino sugar metabolism pathways in *S. pneumoniae* (Kanehisa et al., 2014). The *aga* operon consists of a gene encoding a beta-galactosidase (*bgaC*), a predicted galactosamine disaccharide-specific PTS system (*agaVWEF*), a gene coding for a sugar isomerase (*agaS*) and a gene coding for an aldose 1-epimerase (*galM*). *agaVWEF* is the annotated PTS for the transport of NAGa, whereas the deacetylation of NAGa-6-P and deamination/isomerization of GalN-6-P may be performed by NagA and NagB, respectively. Most of the genes involved in amino sugar metabolism are regulated in our transcriptome analysis in the presence of NAGa, suggesting that the growth conditions used in our studies are adequate and that the NAGa pathway is functional.

One of the important enzymes regulated in our study is BgaC, which is a novel surface-exposed glycohydrolase, which has been shown to have an effect on *S. pneumoniae* adhesion and virulence (Jeong et al., 2009; Terra et al., 2010). A classic β-galactosidase (EC 3.2.1.23) BgaC displays explicit hydrolysis activity toward the terminal Galβ (1,3) NAG moiety of oligosaccharides (Jeong et al., 2009; Terra et al., 2010). Sequence comparison puts BgaC into the glycosidase family 35 (GH-35), which mainly occurs in higher eukaryotes (Henrissat and Davies, 1997). BgaC receives a sequence and structural organization arrangement similar to β-galactosidases from higher eukaryotes and microbial pathogens instead of typical prokaryotic β-galactosidases (Henrissat and Davies, 1997). Several crystal structures of β-galactosidases have been submitted in the Protein Data Bank (PDB) database. These include *E. coli* β-gal (Jacobson et al., 1994), *Arthrobacter* sp. C2-2 C221 β-gal (Skálová et al., 2005), *Kluyveromyces lactis* β-gal (Pereira-Rodríguez et al., 2012), *Thermus thermophilus* A4 β-gal (Hidaka et al., 2002), *Bacillus circulans* sp. *alkalophilus* β-gal (Maksimainen et al., 2012), *Sulfolobus solfataricus* β-gal (Aguilar et al., 1997), *Penicillium* sp. β-gal (Rojas et al., 2004), *Bacteroides thetaiotaomicron* β-gal, 5 *Trichoderma reesei* β-gal (Maksimainen et al., 2011), and *Homo sapiens* β-gal (Ohto et al., 2012). Most of these enzymes possess specific hydrolysis activity toward β (1,4)-galactosyl bond, whereas *H. sapiens* β-gal exhibits hydrolysis activity toward both β(1,3)- and β(1,4)-galactosyl bonds (Alpers, 1969; Asp and Dahlqvist, 1972; Distler and Jourdian, 1973), and the substrate specificity of *B. thetaiotaomicron* β-gal remains unknown. *E. coli* β-gal, as a member of GH-2, is one of the most extensively studied β-galactosidases. The presence of BgaC in *S. pneumoniae* suggests that pneumococcus has the ability to make use of the galactose moieties present in its environment.

In *E. coli*, the *aga* genes are regulated by the transcriptional regulator AgaR, which is a DeoR family transcriptional repressor (Ray and Larson, 2004). AgaR binds in tandem to several repeat sequences in the intergenic regions of *agaZ, agaR*, and *agaS* to repress the transcription (Leyn et al., 2012). In *S. pneumoniae*, the *aga* operon is also regulated by transcriptional factor AgaR. The AgaR present in *S. pneumoniae* belongs to the GntR family of transcriptional repressor. It has a winged helix-turn-helix (WHTH) DNA-binding domain and an UbiC transcription regulator-associated (UTRA) domain. Several bacterial and archaeal genomes possess representatives of GntR family of transcriptional regulators. Numerous biological processes, including antibiotic production, sensing of nutritional status, growth, proliferation, development, diverse metabolic processes (fatty acid metabolism, amino acid metabolism, acetoin utilization, etc.) are controlled by the GntR family regulators (Wiethaus et al., 2008; Resch et al., 2010). In this study, we have identified an AgaR operator site in the P*bgaC*, which was further verified by promotor mutational analyses. To explore more putative AgaR operator sites in the D39 genome, we conducted a genome-wide search with the putative pneumococcal AgaR operator site. AgaR operator site was only found in the promoter region of *bgaC* suggesting the *aga* operon as the only target of AgaR in *S. pneumoniae* D39. This predicted AgaR operator site is also found highly conserved in other streptococci as well (Novichkov et al., 2010), suggesting a similar function of AgaR in other streptococci as well.

The master regulator, CcpA, is involved in the regulation of genes involved in sugar metabolism and has a role in the pathogenesis (Lulko et al., 2007; Zomer et al., 2007; Carvalho et al., 2011). A number of other non-preferred sugar systems are also regulated independently of CcpA like CelR in *S. pneumoniae* (Shafeeq et al., 2011b). The expression of the *aga* operon did not go very high in our transcriptome in the presence of NAGa (Table 3). The moderate upregulation of the *aga* operon in the presence of NAGa suggested the involvement of another transcriptional regulator in the regulation of the *aga* operon. The presence of a *cre* box in the P*bgaC* indicates that CcpA could have a role in the regulation of the *aga* operon, which is further confirmed by our microarray analysis of *S. pneumoniae* D39 Δ*ccpA* against the D39 wild-type. These results are also consistent with the study performed by Carvalho et al. (2011).

## Author contributions

MA designed the project, performed experiments, and wrote manuscript. SS designed the project and wrote the manuscript. HA performed experiments. OK designed the project and wrote manuscript.

### Conflict of interest statement

The authors declare that the research was conducted in the absence of any commercial or financial relationships that could be construed as a potential conflict of interest.
